# The Use of Complementary and Alternative Medicine in Thai Gynecologic Oncology Patients: Influencing Factors

**DOI:** 10.1155/2021/1322390

**Published:** 2021-11-10

**Authors:** Nuntorn Chukasemrat, Chuenkamon Charakorn, Arb-aroon Lertkhachonsuk

**Affiliations:** Division of Gynecologic Oncology, Department of Obstetrics and Gynecology, Faculty of Medicine Ramathibodi Hospital, Mahidol University, Bangkok, Thailand

## Abstract

**Background:**

To determine the factors influencing the use of complementary and alternative medicine (CAM) in gynecologic cancer patients and the prevalence and pattern of CAM use.

**Methods:**

This was a cross-sectional study of 370 gynecologic cancer patients conducted at the outpatient clinic, Ramathibodi Hospital, Mahidol University, Bangkok, Thailand. After obtaining informed consent, participants were asked to complete a standardized questionnaire including sociodemographic and clinical characteristics, detail of CAM use, attitude of CAM use, and quality of life using EORTC-QLQ-C30.

**Results:**

The prevalence of CAM use was 25.13%. The most common type was herbal medicine (55.90%). The participants who resided or had a birthplace in rural areas presented with a higher proportion of CAM use than those in urban areas (*P*=0.470 and *P*=0.004, respectively). Participants who received multiple modalities of cancer treatment reported a significantly higher proportion of CAM use (*P*=0.024). Most CAM users agreed that the CAM could be used in combination with standard treatment, and some rather disagreed that CAM could interrupt the treatment effect of the conventional treatment. CAM users had significantly higher role functioning in quality-of-life scores.

**Conclusion:**

Factors influencing CAM use in gynecologic cancer patients were rural area birthplace or residency, receiving multiple modalities of cancer treatment, having positive attitude toward CAM use. CAM users had better performance in role functioning in the quality-of-life score. Therefore, gynecologic oncologists should pay attention to these factors in order to communicate with gynecologic cancer patients about CAM use.

## 1. Introduction

Cancer is a global leading cause of morbidity and mortality. Despite the standard treatments developed for a better outcome, cancer patients still experience side effects from these treatments [1, 2]. Therefore, there have been some cancer patients who decided to choose the complementary and alternative medicine (CAM) in replacement or combination with the conventional treatment. Horneber and colleagues studied the use of CAM in 18 Western countries and summarized that the prevalence of CAM use increased from 25 to 49% during 1970 and 2000. Moreover, this tendency continued to increase [3]. Mao and colleagues did a survey including 23,393 participants, of which 66.5% of the cancer patients were treated with CAM. Interestingly, they found that most of the patients had breast or gynecological cancer [4].

According to the National Center for Complementary and Integrative Health (2016) [5], CAM is defined as treatment, medication, or practice used together with, or in place of, conventional medical treatment. It is divided into three categories: (1) the application of natural products, for example, herbal drugs, vitamins, or eating habit change (e.g. vegetarian); (2) mind and body practices such as meditation, art and music therapy, aromatherapy, yoga, Tai Chi, or acupuncture; and (3) other methods.

When focusing on gynecological cancer patients, a systematic review including 12 research works revealed that the prevalence of CAM use ranged from 40.4 to 94.7%. CAM use was common in 20- to 30-year-olds, those older than 60 years, or among well-educated persons [6]. Abdallah and colleagues performed a questionnaire survey in 534 gynecological oncology patients. They discovered that 86.9% of relevant patients adopted the CAM use, and the majority of them used natural products, including herbal medicine or vitamin supplement [7]. Ben-Arye and colleagues examined the referral patterns of oncology healthcare providers to an integrative oncology program in which patients with cancer were offered a number of CAM treatment options in Israel. The characteristics of the referral patterns for patients with gynecologic cancer were compared with those for patients with nongynecologic malignancies. It appeared that there was significantly more proportion of gynecological cancer patients who needed the CAM treatment than other type of cancer patients [8].

Considering the factors affecting the CAM use, Molassiotis et al. found that the factors of choosing the CAM for cancer patients in Europe were connected with those who are younger and have higher education [3, 4, 9]. In Thailand, Supoken et al. performed an interview study about the use of CAM in 100 patients newly diagnosed with gynecological cancer within one month, 67 of them were treated with the alternative medicine. Most of them in higher cancer stage (stages 3–4) and received chemotherapy more than other modalities [10]. Then, Puataweepong et al. conducted a survey study at the radiotherapy outpatient unit. They found that the prevalence of CAM use was 60.9%, which was significantly associated with high-income patients [11].

At present, CAM is more popular because there have been several studies suggested its role in strengthening the immune system [4, 7, 11], reducing the side effects of cancer treatment [4, 11, 12], and improving the patients' physical and psychological conditions [9, 13, 14]. On the other hand, some studies reported that CAM interacts with the conventional treatment, causing adverse effects. Drozdoff et al. conducted a questionnaire survey of 448 patients with breast or gynecological cancer. Approximately three-fourths of the respondents declared biological-based CAM use concomitant with systemic cancer therapy. Around one-fifth from the participants' information led to a classification of suspected CAM-drug interaction because the interactions of the combination of CAM and CYP3A4-metabolized anticancer drugs were found in preclinical studies, though not verified with clinical data [15]. Compared with previous studies, Zeller et al. performed an interview study and reported that 64% of their participants used CAM and a third were in danger of CAM-drug interactions [16]. Chotipanich et al. published a research of 426 cancer patients in Thailand who have undergone the conventional treatment and figured out that 45.1% of them chose CAM and 14.4% of them had missed treatment schedules [17].

In Thailand, CAMs such as Thai traditional medicine, Thai herbs, Thai massage, acupuncture, and cannabis were promoted by the Thai government [18]. Thailand has been the first country in Southeast Asia allowing the use of medical cannabis since December 2018. Even though cannabis is still listed as category 5 in narcotic drugs and without enough evidence to support its use in cancer treatment, medical cannabis has been legalized for some purposes in the health care and research. For this reason, the majority of Thai people draw attention to seek the alternative medicine information from the accessible journals and social media.

Because the factors affecting CAM use in cancer patients are varying across time and culture, the researchers are interested in exploration of these factors in the gynecological cancer patients. Moreover, we aimed to determine the prevalence and pattern of CAM use in current situation.

## 2. Materials and Methods

A cross-sectional study was conducted at the outpatient gynecologic oncology clinic at the Faculty of Medicine Ramathibodi Hospital, Mahidol University, Bangkok, Thailand. The ethical approval was accepted by the Human Research Ethics Committee, Faculty of Medicine Ramathibodi Hospital, Mahidol University, on April 13, 2020 (COA. MURA2020/635). All participants who voluntarily decided to join the study were given the study information. Patients were asked to complete a standardized questionnaire. The study took place between May 2020 and January 2021. Written informed consents were obtained from all participants.

### 2.1. Population

The inclusion criteria were Thai patients with gynecologic malignancies, who visited the outpatient clinic. The participants should be at least 18 years old. They needed to be fully conscious, literate, with normal vital signs, and a pain score less than 3. Participants who denied participating the study were excluded.

### 2.2. Questionnaire

A self-administered questionnaire was developed with both open- and closed-ended questions. Factors expected to be relevant with CAM use were derived from literature review. The questionnaire was validated and approved by three experts and then was piloted in 10 patients. The questionnaire is composed of 3 parts, i.e., sociodemographic and clinical characteristics, details and attitude toward CAM use, and quality of life.

The first part of the questionnaire included patients' age, marital status, caregiver, birthplace (urban (defined as Bangkok and surrounding provinces) vs rural area), residency, occupation, education, personal income, family income, family member, religion, performance status by Eastern Cooperative Oncology Group (ECOG) score, health care benefit scheme, and underlying disease. Cancer stage at diagnosis and time since cancer diagnosis, which started from time at diagnosis to time at conducting the survey, were collected. Disease status, current treatment, and modality of treatment were also noted.

The second part of the questionnaire comprised types, duration, expenses, and results regarding CAM use. There was also an open-ended question asking about their opinion regarding CAM use. There were 4 questions asking about attitude toward CAM use, i.e., whether or not CAM use should replace, or be combined with, or interrupt the treatment effect of the standard cancer treatment. The last question concerned about the attitude in the safety of CAM use. Attitude toward CAM use was scaled into 5 levels of agreement (Likert rating scale).

The last part of the questionnaire was the patients' quality of life. We assessed the quality-of-life-related outcomes by using the European Organization for Research and Treatment of Cancer Quality of Life Questionnaire (EORTC-QLQ-C30), which had been validated in Thai language and in the third version [19, 20]. We asked for the permission for the use of this questionnaire. The EORTC-QLQ-C30 was a 30-item questionnaire include a five multiitem functional scale (physical, role, emotional, cognitive, and social function), a three multiitem symptom scale (fatigue, pain, and nausea/vomiting), and six single items assessing further physical symptoms scales (appetite loss, constipation, diarrhea, dyspnea, and insomnia) and financial difficulties [20].

### 2.3. EORTC Scoring Calculation and Interpretation [21]

EORTC scoring was calculated by averaging the items scored (raw score, *R*_S_) using a linear transformation to standardize the *R*_S_ to range from 0 to 100, with *R*_S_ indicating the mean of the component items. Functional scores were calculated using the following equation: Score = {1 − (*R*_S_ − 1)/range} × 100. Symptom scales/items and global health status/QOL were calculated as well: Score = {(*R*_S_ − 1)/range} × 100. Thus, a high score for the global health status represents a better QOL, a high score for a functional scale represents a better or healthy level of functioning, but a high score for a symptom scale represents a worse symptom.

### 2.4. Statistical Analysis

The sample size was estimated by an infinite population proportion formula based on the prevalence of CAM use presented by Puataweepong et al. [11]. With an alpha of 0.05, a number of 366 participants were needed. Demographic characteristics were summarized by descriptive statistics according to the types and distribution of data. For categorical data, the association between interested characteristics and CAM use was determined by chi-square or Fisher's exact test, which is appropriate. For continuous data, Student's *t*-test or Mann–Whitney *U* test was applied according to the distribution of data. A *P* value of 0.05 was considered for statistical significance. The statistical analysis was performed by using STATA version 16 (StataCorp, College Station, Texas, USA).

## 3. Results

From May 2020 to January 2021, a total of 408 patients were enrolled; 38 of them were excluded due to their denial to participate in the study or incomplete questionnaire answer. So, a total of 370 questionnaires were retrieved for data analysis. The mean age of participants was 55.1 years (ranged from 18 to 89 years). Most of them were married (50.8%), had rural birthplace (64.6%), and had a Bachelor degree (45.7%). Half of them had personal monthly income of more than 15,000 Baht. A vast majority (96.7%) were of Buddhism background. The diagnoses were ovarian, fallopian tube, or primary peritoneal cancer 142 (38.38%); uterine cancer 112 (30.27%); cervical cancer 88 (23.78%); gestational trophoblastic neoplasia 15 (4.05%); vulva cancer 7 (1.89%); and other gynecologic cancer 6 (1.62). Most of the patients were diagnosed with cancer in early stage (stage I or II) (56.8%). The common combination treatment was between surgery and chemotherapy (36.8%). Common current cancer status was in remission (60.5%).

Ninety-three participants (25.13%) reported an experience of CAM use at least once in their lifetime. History of CAM use and sociodemographic characteristics were compared between the 2 groups as shown in Table 1. No statistical differences between the two groups were observed for the following factors: age, marital status, caregiver, occupation, education, personal income, family income, religion, underlying disease, ECOG score, and health care benefit scheme. However, the participants with rural area of birthplace or residency had a higher proportion of CAM use than those born or residing in the urban area (*P*=0.47 and *P*=0.004, respectively). Considering the clinical characteristics demonstrated in Table 2, there was no statistically significant difference between cancer stage, time since diagnosis, disease status, or current treatment and CAM use. Nevertheless, those with multiple modalities of treatment were more likely to use CAM (*P*=0.024).

Table 3 presents the types of CAM use in our participants. There were participants using more than one CAM type. The most common type of CAM use was herbal medicine (55.9%) with cannabis as the most frequently used (18.28%). Sixty-three percent of CAM users were still using CAM even though the standard treatment had ended. Seventy-four participants decided to use CAM when they have been diagnosed with cancer. Forty-eight users (51.6%) believed that CAM would have the antitumor effect. Besides, the other reasons for CAM use were the beliefs that CAM should improve mental condition (47.3%), improve physical condition (45.2%), prevent cancer recurrence (31.2%), control pain (20.4%), and improve nutritional status (7.5%). Half of the CAM use (50.5%) paid less than 1,000 Baht per month, while 14% paid more than 6,000 Baht per month. Sixty participants (64.5%) did not inform their physicians about the CAM use. The reasons were disapproval of CAM use by their physicians (82.3%), belief of unaffected effect between CAM and the standard treatment (70.6%), and concern about their physicians' dissatisfaction (11.8%).

However, when inquired about overall opinion, both CAM (81.7%) and non-CAM (96.93%) users agreed that their physician should be informed. Unsurprisingly, those who disagreed to tell their physician about CAM use were significantly associated with the CAM users with *P* valve < 0.001. The reasons for participants who informed their physician about CAM usage were the need of physicians' consideration between CAM use and conventional treatment (78.4%). Moreover, some of them wanted to know their physician's opinion on their CAM use (30.5%).

The attitude of participants toward CAM use is presented in Table 4. Although most of the participants rather disagreed that CAM could replace the conventional medicine, CAM users also agreed that the CAM could be used in combination with the standard treatment. While the CAM users rather disagreed that CAM could interrupt the conventional treatment, non-CAM users agreed with this. Regarding the factors affecting the quality of life, as shown in Table 5, it was found that, in statistics, the participants adhered to CAM use had significantly better quality of life in dimension of work and daily activities (as demonstrated in the role functioning item of the functional scale).

## 4. Discussion

In this study, only a quarter of our patients with gynecological cancer experienced CAM use. This was lower than the prevalence in precedent research works conducted in China, Australia, America, and Europe, which stated that 40% of patients with gynecological cancer adopted the alternative medicine [3, 22–25]. To compare with studies conducted ten years ago in Thailand, the prevalence of CAM use was reported around 60% [10, 11]. The prevalence in Asia is more obvious than that in America or Europe [26]. However, there were quite a few studies regarding CAM use and cancer patients across the Asian countries. Types of CAM use such as nature products and herbs were similar to the previous studies. Furthermore, it appeared that the use of cannabis is more mentioned in our research due to the fact that, in Thailand, cannabis has been legalized for medical purposes since 2018, drawing the attention of Thai people to seek for CAM use.

In a large study conducted in 32 countries, including Asia, it was determined that the following basic sociodemographic and health data were correlated with the choice of CAM, i.e., gynecological cancer, younger women, persons with higher education, and income [27]. According to our study, the factors affecting CAM use were irrespective of age, education, occupation, and income, but associated with rural birthplace and residence. The residence and birthplace area were factors influencing the patients' treatment, possibly due to the different infrastructure of local communities and culture influences, which played a role in determining the people's belief and in formulating the health promotion practice and modern medical treatment. There was a cross-sectional study assessing CAM use among Malaysian population; a higher frequency of CAM use among different cultural groups was obviously indicated. For instance, massage was the usual practice in Malay community, while acupuncture was widespread in Chinese community [28]. Meanwhile, there was a study observing 1,427 Australians that specified that those who lived in rural areas used more CAM than those who lived in urban areas [29]. So, this evidence supported that CAM use was more common in rural areas by the reason of the difficulty to access health care. The cultural influence in rural community may result in this as well.

The use of CAM in this research was not associated with other clinical factors, except with patients undergoing multimodalities cancer treatment. These patients might be more likely to develop treatment complications and side effects. Ben-Arye et al. found that CAM use can help alleviating the toxicity of chemotherapy 28.8%, reducing fatigue from chemotherapy 17.9%, and relieving emotional stress 7.1% [8]. As mentioned in our study, most of the patients believed that CAM could prevent cancer and improve mental and physical conditions. Other research also reported the other advantages of CAM in aspect of boosting immune system, reducing chemotherapy side effects, and relieving stresses in their body and mind [4, 5, 8, 9, 11, 12, 26].

In relation to the attitude toward CAM use, most participants rather disagreed that CAM could interrupt the conventional medicine. In comparison, a higher proportion of CAM users strongly disagreed with this attitude than non-CAM users. However, recent studies discover that many types of CAM interact with the conventional treatment. Especially, natural products or biological-based compounds have mechanisms via which these interactions may occur, as divided into pharmacokinetics and pharmacodynamics. On the pharmacokinetics effect, enzymes of the cytochrome P450 (CYP450) family and membrane transporters such as P-glycoprotein play important roles in the absorption and metabolism of many prescription drugs. Herbal medicines such as ginseng, gingko, turmeric, and *Echinacea* inhibition or induction of CYP450 enzymes influence on the metabolism of chemotherapy, e.g., cyclophosphamide, paclitaxel, doxorubicin, or irinotecan. This may lead to subtherapeutic drug levels as well as to prolonged activity and even toxicity of a drug [14, 30]. On the pharmacodynamics effect, antioxidant supplements could interfere with radiation or with any chemotherapy that operates via a free radical mechanism [31, 32]. For instance, vitamin E and beta-carotene reduce toxicity from radiotherapy among patients with head and neck cancer, and hence it has been found to increase recurrence [33]. In addition, a report in Thailand revealed another disadvantage of CAM use in their study, 14.4% of CAM users missed the treatment schedule for conventional treatment [17].

When focusing on the quality of life, we found that CAM users had higher role functioning in the quality-of-life scale than non-CAM users. Few research works have evaluated the effect of CAM use in an aspect of quality of life in symptomatic gynecological cancer patients.

More than 90% of participants agreed that they should inform their physician when they made a decision on CAM use. However, precedent studies reported similar opinion in only 60% [10]. In addition, the reason that they did not inform their physicians about CAM use was the physicians did not ask them. As previously mentioned, natural products of CAM may affect the standard treatment effects and loss of treatment schedule. Therefore, the oncologists should be aware of this issue.

One of the strengths in this study is the specified population in gynecologic cancer patients. Moreover, this is the first few studies in recent years aimed to assess factors influencing the use of CAM, including the views of demographic, disease, attitude, and quality of life. Although the survey was conducted in a single university hospital, the patients were referred from various parts of Thailand and might represent the general Thai cancer population. The limitations of our study were the majority of our participants' disease status was inactive and the limited number in palliative setting.

The factors influencing the use of CAM in gynecological cancer patients are the rural birthplace and residency, receiving multiple modalities of treatments, and positive attitude toward CAM use. CAM users had better role functioning in the quality-of-life score. A quarter of participants have ever used CAM treatment at least once in a lifetime and herbal medicine is more popular than others. There has been evidence-based information of CAM in either support or interact with conventional treatment. Therefore, gynecologic oncologists and other health care providers who get involved in the gynecologic cancer patients care should be aware of, inquire about, and pay attention to the CAM use, especially in patients with previously mentioned characteristics, in order to counsel the patients about the risks and benefits of CAM use alongside conventional treatment. A further study should be conducted in patients with active disease or in palliative setting.

## Figures and Tables

**Table 1:** Sociodemographic characteristics between complementary and alternative medicine users and nonusers.

Sociodemographic characteristics	CAM users, *n* = 93 (%)	Non-CAM users, *n* = 277 (%)	*P* value
Mean age (mean ± SD)	53.48 ± 10.89	55.67 ± 14.26	0.124
Marital status			
Pair/Married	50 (54.35)	138 (50.36)	0.574
Divorced/separated/widowed	21 (22.83)	58 (21.17)	
Single	21 (22.83)	78 (28.47)	

Care giver			
Parent/descendant/partner	66 (70.97)	211 (76.17)	0.390
Relatives/nursing home/friend	18 (19.35)	50 (18.05)	
None	9 (9.68)	16 (5.78)	

Birthplace			
Urban	25 (26.88)	106 (38.27)	0.047
Rural	68 (73.12)	171 (61.73)	

Residence			
Urban	42 (45.16)	172 (62.09)	0.004
Rural	51 (54.84)	105 (37.91)	

Occupation			
Unemployed	30 (32.26)	95 (34.30)	0.719
Employed	63 (67.74)	182 (65.70)	

Education			
Secondary school	45 (48.39)	117 (42.24)	0.301
Higher level than secondary school	48 (51.61)	160 (57.76)	

Personal income (Baht/month)			
≤15,000	38 (44.19)	106 (40.61)	0.560
>15,000	48 (55.81)	155 (59.39)	

Family number (person)			
≤3	54 (58.06)	157 (56.68)	0.815
>3	39 (41.94)	120 (43.32)	

Family income (Baht/month)			
≤15,000	27 (18.3)	37 (13.4)	0.245
>15,000	76 (81.7)	240 (86.6)	

Religion			
Buddhism	90 (96.8)	268 (96.8)	0.369
Muslim	1 (1.1)	7(2.5)	
Christianity	2 (2.2)	2(0.7)	

Underlying disease			
No	47 (50.54)	149 (53.79)	0.587
Yes	46 (49.46)	128 (46.21)	

ECOG score			
0	82 (88.17)	254 (91.7)	0.595
1	10 (10.75)	21 (7.58)	
2	1 (1.08)	22 (1)	

Health care benefit scheme			
Civil service welfare	43 (46.24)	135 (48.74)	0.989
Social security	7 (7.53)	25 (9.03)	
Universal coverage	21 (22.58)	57 (20.58)	
Others	22 (23.65)	60 (21.65)	

CAM, complementary and alternative medicine; ECOG, Eastern Cooperative Oncology Group.

**Table 2:** Clinical characteristics between complementary and alternative medicine users and nonusers.

Clinical characteristic	CAM users, *n* = 93 (%)	Non-CAM users, *n* = 277 (%)	*P* value
Cancer stage			
Stage I, II	48 (51.61)	162 (58.48)	0.102
Stage III, IV	45 (48.38)	108 (38.99)	

Time since cancer diagnosis			
<1 year	40 (44.44%)	138 (51.30%)	0.279
1–2 year	18 (20%)	59 (21.93%)	
>2 year	32 (35.56%)	72 (26.77%)	

Modality treatment combination			
Single modality	29 (31.18)	83 (31.32)	0.024
Two modalities	47 (50.54)	160 (60.32)	
Three modalities	17 (18.28)	22 (8.3)	

Disease status			
Remission	63 (67.74)	161 (58.12)	0.637
Stable	17 (18.28)	72 (25.99)	
Recurrent	6 (6.45)	25 (9.03)	
Progressive disease	2 (2.15)	19 (6.86)	

Current treatment			
No treatment, surveillance	60 (64.52)	158 (57.04)	0.333
Chemotherapy	24 (25.81)	93 (34.30)	
Surgery	7 (7.53)	13 (4.69)	
RT/CCRT	1 (1.08)	9 (3.25)	
Best supportive care	1 (1.08)	2 (0.72)	

CAM, complementary and alternative Medicine; RT/CCRT, radiation therapy/concurrent chemotherapy with radiation treatment.

**Table 3:** Types of complementary and alternative medicine used.

Natural products		Frequency (%)
Herbal medicine		52 (55.9)
	(i) Cannabis	17 (18.28)
	(ii) Ginger	15 (16.13)
	(iii) Turmeric	13 (13.98)
	(iv) Garlic	7 (7.53)
	(v) Ginseng	2 (2.15)
	(vi) Lingzhi mushroom	2 (2.15)
	(vii) Crocodile blood	2 (2.15)
	(viii) Other	5 (5.37)

Diet adjustment		41 (44.1)
	(i) Vegetarian food	15 (16.13)
	(ii) Bio-organic food	9 (9.68)
	(iii) Vitamin	19 (20.43)
	(iv) Other	22 (23.65)

Mind and body practices		
	Pray and meditation	49 (52.69)
	Massage	11 (11.83)
	Naturopathy	10 (10.75)
	Yoga	8 (8.60)
	Detoxification	5 (5.38)
	Hypnosis	5 (5.38)
	Homeopathy	3 (3.22)
	Aromatherapy	1 (1.07)

**Table 4:** Attitude toward complementary and alternative medicine use between users and nonusers.

		CAM users	Non-CAM users	*P* value
CAM could replace conventional medicine				
	(i) Strongly disagree	21(22.58)	61(22.02)	0.911
	(ii) Rather disagree	33(35.48)	154(55.60)	0.001
	(iii) Agree	26(27.96)	53(19.13)	0.072
	(iv) Rather agree	10(10.75)	7(2.53)	0.001
	(v) Strongly agree	3(3.23)	2(3.7)	0.070

CAM could be combined with conventional medicine				
	(i) Strongly disagree	3(3.23)	22(7.94)	0.117
	(ii) Rather disagree	10(10.75)	59(21.38)	0.024
	(iii) Agree	42(45.16)	146(52.71)	0.208
	(iv) Rather agree	21(22.58)	32(11.55)	0.009
	(v) Strongly agree	17(8.8)	18(6.50)	0.001

CAM could interrupt conventional treatment				
	(i) Strongly disagree	23(24.73)	36(13)	0.007
	(ii) Rather disagree	47(50.54)	142(51.26)	0.904
	(iii) Agree	17(18.28)	80(28.88)	0.044
	(iv) Rather agree	3(3.23)	16(5.78)	0.335
	(v) Strongly agree	3(1.5)	3(1.00)	0.157

CAM is not safe				
	(i) Strongly disagree	5(5.38)	13(4.69)	0.791
	(ii) Rather disagree	32(34.41)	80(28.88)	0.315
	(iii) Agree	39(41.94)	127(45.85)	0.512
	(iv) Rather agree	12(12.90)	35(12.64)	0.946
	(v) Strongly agree	5(5.38)	22(7.94)	0.410

CAM, complementary and alternative medicine.

**Table 5:** Quality of life between complementary and alternative medicine users and nonusers.

EORCTC QLQ-30	CAM users	Non-CAM users	*P* value
Global health status/QOL; mean ± SD	68.91 ± 20.19	68.38 ± 19.67	0.824
Functional scale; mean ± SD			
Physical functioning	83.15 ± 16.39	82.55 ± 18.93	0.784
Role functioning	90.14 ± 14.99	85.19 ± 20.94	0.036
Cognitive functioning	82.44 ± 15.99	83.51 ± 17.92	0.607
Emotional functioning	77.78 ± 18.65	77.71 ± 17.91	0.974
Social functioning	84.05 ± 19.18	82.37 ± 21.49	0.504

Symptom scales/items; median (IQR)			
Fatigue	33.33 (22.22–44.44)	33.33 (22.22–44.44)	0.699
Nausea and vomiting	0 (0–0)	0 (0–16.67)	0.549
Pain	16.67 (0–33.33)	16.67 (0–33.33)	0.662
Dyspnea	0 (0–33.33)	0 (0–33.33)	0.405
Insomnia	33.33 (0–33.33)	33.33 (0–33.33)	0.786
Appetite loss	0 (0–33.33)	0 (0–33.33)	0.759
Constipation	0 (0–33.33)	33.33 (0–33.33)	0.377
Diarrhea	0 (0–0)	0 (0–0)	0.958
Financial difficult	33.33 (0–33.33)	0 (0–33.33)	0.078

EORTC-QLQ-30, European Organization for Research and Treatment of Cancer Quality of Life Questionnaire 30 items; IQR, interquartile range; SD, standard deviation.

## Data Availability

The data that support the findings of this study are available from the corresponding author upon reasonable request.
